# Transcriptome and ultrastructural changes in dystrophic Epidermolysis bullosa resemble skin aging

**DOI:** 10.18632/aging.100755

**Published:** 2015-06-14

**Authors:** Jenny S. Breitenbach, Mark Rinnerthaler, Andrea Trost, Manuela Weber, Alfred Klausegger, Christina Gruber, Daniela Bruckner, Herbert A. Reitsamer, Johann W. Bauer, Michael Breitenbach

**Affiliations:** ^1^ Department of Dermatology and EB House Austria, Paracelsus Medical University, Salzburg, Austria; ^2^ Fachbereich Zellbiologie der Universität Salzburg, Salzburg, Austria; ^3^ University Clinic of Ophthalmology and Optometry, Research Program for Ophthalmology and Glaucoma Research, Paracelsus Medical University, Salzburg, Austria

**Keywords:** microarray analysis of skin aging, bullous skin disease, immune fluorescence microscopy, loricrin, keratin, collagen

## Abstract

The aging process of skin has been investigated recently with respect to mitochondrial function and oxidative stress. We have here observed striking phenotypic and clinical similarity between skin aging and recessive dystrophic Epidermolysis bullosa (RDEB), which is caused by recessive mutations in the gene coding for collagen VII, *COL7A1*. Ultrastructural changes, defects in wound healing, and inflammation markers are in part shared with aged skin. We have here compared the skin transcriptomes of young adults suffering from RDEB with that of sex‐ and age‐matched healthy probands. In parallel we have compared the skin transcriptome of healthy young adults with that of elderly healthy donors. Quite surprisingly, there was a large overlap of the two gene lists that concerned a limited number of functional protein families. Most prominent among the proteins found are a number of proteins of the cornified envelope or proteins mechanistically involved in cornification and other skin proteins. Further, the overlap list contains a large number of genes with a known role in inflammation. We are documenting some of the most prominent ultrastructural and protein changes by immunofluorescence analysis of skin sections from patients, old individuals, and healthy controls.

## INTRODUCTION

Some of the skin phenotypes of RDEB patients clinically resemble the phenotypes seen in elderly individuals (Fine 2014). We have started the present investigation in order to determine if these similarities are only superficial or might be based on parallel and similar gene expression changes. The methods used were whole genome transcription studies using microarrays as well as analysis of selected skin proteins by immuno-fluorescent staining of skin sections. The skin problems found in RDEB patients include as primary lesions blisters and erosions, dystrophy or absence of nails, alopecia, exuberant granulation tissue (in junctional EB), congenital absence of skin, palmoplantar keratoderma, mottled pigmentation (EB simplex) and characteristic nevi [1]. The primary lesions are typical for EB and are not seen in intrinsic skin aging. However, the secondary changes in EB include atrophies, scarring and pigmentary abnormalities. These abnormalities can be also seen in aging skin. Atrophy can be characterized in aging skin by fine wrinkling or flattening of epidermal ridges. This is mirrored on the ultrastructural level by thinning of the epidermis and a loss of collagen fibers. In addition, in EB there is a loss of skin appendages like sweat glands and sebaceous glands through scarring. A loss of these glands can also be seen in skin aging caused by involution. Most importantly, the essential barrier function of skin is (partially) compromised in both conditions [2–4] causing a number of pathological consequences like dry and itchy skin, an increased risk of skin superficial infections, for instance fungal infections, and defects of wound healing. These are hallmarks of intrinsic skin aging in non-sun-exposed skin. The barrier function of skin relies on the density and the hydrophobic nature of the top layer of the epidermis, the cornified envelope (CE). The alterations occurring both in RDEB and skin aging described above are hallmarks of intrinsic skin aging in non-sun-exposed skin. We are showing here transcriptomic and ultra-structural changes in aged and EB skin which are in line and could explain the common phenotypes of the two conditions, including loss of barrier function of the skin.

## MATERIALS AND METHODS

### 1 Skin tissue and patient material

#### 1.1 RDEB patients and healthy probands

Skin samples were taken with informed consent during the course of medically necessary surgical treatments. Skin samples of three male young adult RDEB patients were used for RNA preparation and for immune fluorescence microscopy. Additionally three age and sex-matched healthy probands donated control skin samples. Information about the patients and controls is shown in the following:

##### Young adult RDEB patients

Patient RDEB1: male, 35a, the patient is a compound heterozygote presenting with mutations 8440 C ➔ T (R2814X) and c.4048-1G ➔ T (acceptor splice site in intron 34); skin sample from right lower leg („normal“ skin); in immuno fluorescence microscopy, type VII collagen is absent using the monoclonal mouse anti collagen VII antibody.

Patient RDEB2: male, 25a, the patient is homozygous for 8440 C ➔ T (R2814X); in immuno fluorescence microscopy type VII collagen is absent using the monoclonal mouse anti collagen VII antibody, however when using the polyclonal rabbit anti collagen VII antiserum, a faint collagen VII protein expression is detectable at the epidermal/dermal junction.

Patient RDEB3: male, 17a, the patient is homozygous for the splice site mutation 976+4A ➔ C in intron 7 leading to absence of splicing and a premature chain termination 34 amino acids later; in immuno fluorescence microscopy type VII collagen is absent using the monoclonal mouse anti collagen VII antibody.

##### Age and sex-matched healthy control skin

Healthy proband1: male 35a, skin sample from abdomen; Healthy proband2: male 35a, skin sample from axilla; Healthy proband3: male 27a, foreskin (not inflamed, no phimosis, removed for cosmetic reasons).

#### 1.2 Skin samples for the comparison between adult (middle aged) and elderly probands

##### Young adult skin tissue

Four healthy probands (young adults) donated non-inflammed foreskin tissue. The samples were either taken because of phimosis (but not in an inflammatory state) or for cosmetic reasons. The age of the four probands in the group of young adults, here called „middle-aged“, was 28 years, 28 years, 23 years and 18 years.

##### Aged adult skin tissue

For the group of elderly men five probands were available, donating non-inflammed foreskin tissue: 74 years, 58 years, 65 years, 61 years and and 71 years old.

### 2 RNA Isolation, whole-genome array analysis of mRNA expression

For the middle-aged/old transcriptome analysis, fresh or deep-frozen skin tissue was homogenized using the ULTRA-TURRAX T8 (IKA) in buffer RLT (Quiagen Valencia, California). Total RNA was afterwards isolated using the RNeasy Midi Kit (Quiagen, Valencia, California). The integrity of RNA was visualized using a denaturing 1% agarose gel containing 2.5% formaldehyde by comparison of the 28S/18S rRNA ratio and in addition by the 260/280 nm absorptivity ratio. Concentration of the RNA was evaluated by the absorbance at 260 nm using the nanodrop ND-1000 (Peqlab, Germany). Whole genome expression analysis was performed by MFTServices/Medical Genetics (Tübingen, http://www.mftservices.de) using the GeneChip HG-U133 Plus2.0 arrays (Affymetrix, Santa Clara, CA). Data were analyzed using the GeneSifter® Analysis Edition (Geospiza/Perkin Elmer).

Skin samples for the comparison of gene expression between RDEB and healthy skin were treated and analyzed in a similar way. Briefly, total skin was homogenized with a tissue tearor (Bio Spec Products, Inc., Bartlesville, OK, USA) in RLT buffer, and total RNA was isolated using the RNEasy Lipid Tissue Mini Kit or the RNEasy Mini Kit (Qiagen, Hilden, Germany). The integrity of RNA was analyzed by estimating the 28S/18S rRNA ratio by electrophoresis on a 1% agarose gel and by the 260/280nm ratio. Whole genome gene expression analysis was performed by KFB (Regensburg, Germany) using the Gene Chip HG-U133 Plus 2.0 Arrays (Affymetrix).

### 3 Antigen mapping by immunofluorescence microscopy

Frozen skin tissue was mounted in a cryostat (HM 550, Microm, Walldorf, Germany) and cross sections of 12 μm were collected on adhesion slides (Superfrost Plus; Thermo Scientific, Wien, Austria) and air-dried for 1 hour at RT. After a 5 min rinse in TRIS-buffered saline (TBS; Roth, Karlsruhe, Germany) sections were fixed in phosphate buffered saline (PBS) containing 4% *para*-formaldehyde (PFA) for 15 min. After three 5 min rinses in TBS, sections were incubated for 1 hour at RT in TBS containing 5% donkey serum (Sigma-Aldrich, Wien, Austria), 1% BSA (Sigma-Aldrich), and 0.5% Triton X-100 (Merck, Darmstadt, Germany). After a 5 min rinse, slides were incubated for triple staining with mouse anti human collagen VII (1:100, Millipore MAB 4315, monoclonal), rabbit anti human Loricrin (1:500, Covance PRB-145P, polyclonal serum) and goat anti human Cytokeratin 16 (C-12) (1:100, Santa Cruz sc-49176, polyclonal serum) in TBS/BSA/TritonX-100 over night at RT. Additionally a polyclonal rabbit anti collagen VII antibody (1:500, Abcam, ab93350) was used to detect fragmented or shortened collagen VII proteins. After a rinse in TBS (three times, 5 min) binding sites of primary antibodies were visualized by corresponding AF488, AF555 and AF647 tagged antisera (1:1000; Invitrogen, Karlsruhe, Germany) in TBS, containing 1% BSA and 0.5% Triton X-100 (1 hour at RT) followed by another rinse in TBS (three times, 5 min). Slides were incubated 10 min with 4′,6-Diamidino-2 phenylindol dihydrochloride (DAPI) (1:4000, stock 1 mg/ml, VWR, Vienna, Austria), rinsed three times 5 min in PBS and were embedded in TBS:glycerol (1:1 at pH 8.6).

A confocal laser scanning unit (Axio Observer Z1 attached to LSM710, Zeiss, Göttingen, Germany; × 20 dry or × 40 and × 60 oil immersion objective lenses, with numeric apertures 0.8, 1.30, and 1.4, respectively) was used to document immunohistochemistry. Sections were imaged using the appropriate filter settings for AF488 (495nm excitation), AF555 (555nm excitation), AF647 (650nm excitation) and DAPI (345nm excitation) and up to four channels were detected and merged electronically. All figures presented were recorded in single optical section mode.

### 4 Semi-quantitative analysis of Loricrin expression in the cornified envelope

To assess the differences in loricrin expression in the cornified envelope in middle-aged, aged and RDEB epidermis, particle size measurement was applied. Therefore loricrin positive structures were quantified using the particle size measurement tool of Image J 1.47v (National Institutes of Health, USA). Four confocal images of each patient (27a, 84a, RDEB, respectively) were analyzed: pictures were converted into RGB (red green blue), the contrast enhanced and the median filter applied to reduce background. To analyze the loricrin particle size, the threshold was adjusted to cover all positive loricrin signals. The epidermis was defined as region of interest and the overlay (= particles in the epidermis) was analyzed and reported as % of area. Differences in particle size measurement of the three patients were calculated by One-way ANOVA using SigmaPlot 12.0 (Systat Software Inc.).

### 5 Bioinformatics and statistics

Biological replicas consisted of the three RDEB patients and three healthy probands in the dEB experiment, and of four middle-aged and five elderly probands in the aging experiment.

After GC-RMA normalization of the microarray data statistical analysis was performed with Welch's t-test and the false discovery rate was evaluated according to Benjamini and Hochberg. Design and analyses of the microarray experiments were performed according to the MIAME guidelines [5].

## RESULTS AND DISCUSSION

### Transcriptome comparisons

First, we want to explain how the transcriptome comparisons discussed in the main part of this paper were performed and calculated and how the gene lists shown in Table I and SI Table I were constructed.

In the case of the RDEB/healthy comparison the threshold for significance was set at a two-fold down- or up-regulation. As data from only three patients could be compared, the Benjamini-Hochberg formalism was not used in this case. Therefore genes over- or under-expressed at least two-fold in all three patients were included in the list. The value given in Table 1 is the mean of the fold over- or under-expression value. In the case of the comparison of adult skin with old skin, where 4–5 probands were in each group, more stringent criteria according to Benjamini-Hochberg could be used.

**Table 1:** List of genes differentially expressed in aging and RDEB The table contains 47 genes which are differentially expressed in skin aging and in RDEB. All the genes contained in this list are discussed in detail in the text. For a complete list of the 261 differentially expressed genes, see the SI Table I.

		fold change		
		microarray		RT PCR
geneID	gene name	control	middle aged	middle aged
		RDEB	old	old
**downregulated**				
**CST6**	Cystatin E/M	**2, 63**	**5, 49**	
**FLG**	filaggrin	**6, 78**	**3, 83**	**2,73 ± 0,02**
**FLG2**	Filaggrin family member 2	**18, 3**	**5, 47**	
**IL1F7**	Interleukin 1 family, member 7 (zeta)	**3, 07**	**9, 97**	
**IL1R2**	interleukin 1 receptor, type II	**2, 79**	**2, 15**	
**IL22RA1**	Interleukin 22 receptor, alpha 1	**2, 4**	**2, 45**	
**KRT2**	Keratin 2	**6, 45**	**13, 23**	
**KRT77**	Keratin 77	**9, 45**	**24, 82**	
**LAMB4**	laminin, beta 4	**2, 2**	**10, 8**	
**LCE1B**	Late cornified envelope 1B	**3, 56**	**9, 07**	
**LOR**	Loricrin	**20, 9**	**5, 74**	**2,53 ± 0,06**
**MST1**	macrophage stimulating 1 (hepatocyte growth factor-like)	**3, 08**	**2, 05**	
**MSTP9**	Macrophage stimulating, pseudogene 9	**3, 43**	**2, 73**	
**NFATC2**	Nuclear factor of activated T-cells, cytoplasmic,calcineurin-dependent 2	**2, 17**	**2, 29**	
**TNFRSF19**	Tumor necrosis factor receptor superfamily, member 19	**2, 49**	**4, 89**	
**upregulated**				
**CCL2**	Chemokine (C-C motif) ligand 2	**2, 3**	**2, 93**	
**CCL5**	Chemokine (C-C motif) ligand 5	**3, 51**	**6, 47**	
**CD47**	CD47 molecule	**3, 07**	**2, 25**	
**CFI**	Complement factor I	**3, 43**	**3, 92**	
**COL13A1**	Collagen, type XIII, alpha 1	**2, 43**	**2, 19**	
**COL1A1**	Collagen, type I, alpha 1	**3, 7**	**9, 1**	
**COL4A2**	Collagen, type IV, alpha 2	**4, 92**	**2, 86**	
**COL6A3**	Collagen, type VI, alpha 3	**3, 19**	**2, 54**	
**CXCL12**	Chemokine (C-X-C motif) ligand 12 (stromal cell-derivedfactor 1)	**2, 59**	**2, 59**	
**CXCL13**	Chemokine (C-X-C motif) ligand 13	**2, 2**	**3, 23**	
**DEFB124**	Defensin, beta 124	**2, 76**	**3, 93**	
**IFI27**	Interferon, alpha-inducible protein 27	**6, 01**	**10, 37**	
**IFI44**	Interferon-induced protein 44	**15, 03**	**6, 64**	
**IFI44L**	Interferon-induced protein 44-like	**2, 94**	**3, 95**	
**IFIH1**	Interferon induced with helicase C domain 1	**2, 14**	**2, 02**	
**IFIT3**	Interferon-induced protein with tetratricopeptide repeats 3	**3, 18**	**2, 36**	
**IFNGR1**	Interferon gamma receptor 1	**4, 56**	**2, 18**	
**IL13RA1**	Interleukin 13 receptor, alpha 1	**2, 52**	**2, 7**	
**IL4R**	Interleukin 4 receptor	**2, 44**	**3, 12**	
**IL8**	Interleukin 8	**16, 09**	**5, 06**	
**ITGB6**	Integrin, beta 6	**2, 94**	**3, 92**	
**KRT16**	Keratin 16	**12, 06**	**13, 28**	
**KRT6A**	Keratin 6A	**3, 85**	**8, 72**	
**KRT6B**	Keratin 6B	**3, 42**	**5, 84**	
**S100A12**	S100 calcium binding protein A12	**3, 39**	**101, 12**	
**S100A7**	S100 calcium binding protein A7	**5, 57**	**10, 63**	**1,87±0,23**
**S100A7A**	S100 calcium binding protein A7A	**2, 85**	**34, 79**	
**S100A8**	S100 calcium binding protein A8	**2, 83**	**16, 62**	**2,65 ± 0,57**
**S100A9**	S100 calcium binding protein A9	**7, 1**	**39, 21**	**2,14 ± 0,43**
**SPRR1A**	Small proline-rich protein 1A	**2, 83**	**2, 85**	**1,62 ± 0,24**
**SPRR2D**	Small proline-rich protein 2D	**4, 09**	**6, 24**	**1,91 ± 0,33**
**SPRR2G**	Small proline-rich protein 2G	**8, 81**	**2, 75**	

We argued that the middle-aged/elderly comparison was the best basis for the aging/dEB comparison, for the following reasons: i) the RDEB patients and probands were also young adults; ii) gene expression patterns in skin before puberty were expected to be quite different to those of grown-ups due to the different hormonal and general physiological status and we wanted to look specifically to aging rather than to maturation during puberty.

**Figure 1:**
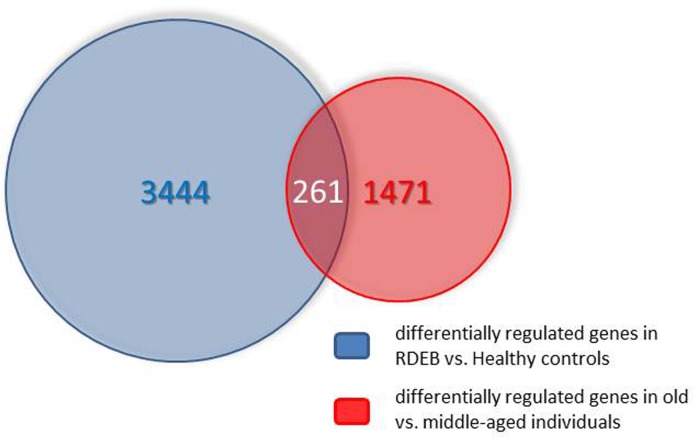
Venn diagram showing the overlap of the transcripts differentially expressed in skin aging (middle aged vs. elderly probands) and in RDEB (RDEB patients vs. age and sex-matched healthy controls). For further explanations see text.

The number of genes (including different known splicing variants derived from individual genes) on the microarray is 38,400. Only a relatively small fraction (978) was found in the middle aged/elderly difference list and in the RDEB/healthy difference list (2447). Comparing these two groups, an overlap list (SI Table I) containing 261 genes was detected. The criterion for inclusion in the list was i) at least two-fold under- or overexpression in both situations compared, and ii) under- or over-expression in the same direction in both situations. The Venn diagram of Fig.1 visualizes these numbers. The probability to find these 261 genes was calculated with an online available tool (http://nemates.org/MA/progs/overlap_stats.html) resulting in a representation factor of 4.2 with a p-value of < 2.209e^−93^, excluding the possibility that the genes in the overlap were found by chance.

Moreover, looking for GO terms in this list does not indicate a random selection of genes but leads to a limited number of functional classes of genes which can be meaningfully correlated with the physiological status of skin in both the RDEB diseased skin and the skin of healthy elderly, as will be discussed in some detail below. Taken together, both arguments show that the method used in all likelihood can inform us about the pathomechanisms acting in both aging and RDEB.

48 of the 261 genes on the overlap list (SI Table I) were selected for the detailed discussion in this study (Table I). These genes (transcripts) were selected on the basis of previously known functions in skin or in the immune system, considering the defense and barrier function of the skin as an immune organ.

### Ultrastructural phenotypes of the skin samples used

It is important that in all three patients, the mutations in the *COL7A1* gene were either premature stop codons or a splicing defect. In all three cases the mutation led to absence of the protein from skin based on the mouse monoclonal antibody mentioned above, as shown by immuno fluorescence microscopy of skin samples. However, comparing transcript levels of *COL7A1* in the RDEB group with the healthy controls showed 2.91-fold overexpression at the RNA level in the patients. This is at variance with the often found nonsense-mediated mRNA decay observed in higher eukaryotic cells.

**Figure 2:**
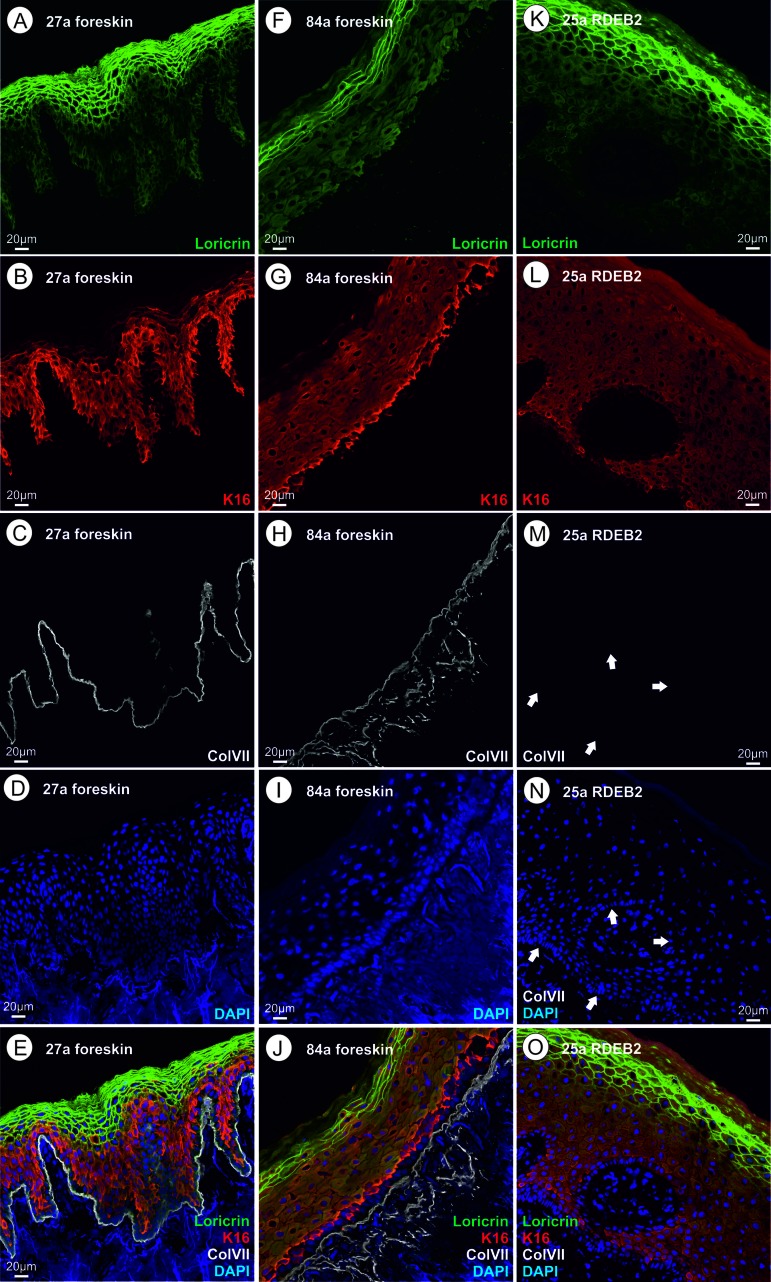
**(A‐E)** skin thin section of a healthy 27 year old proband; A loricrin immune fluorescence; B keratin XVI immune fluoresecence; C collagen VII immune fluorescence using a mouse monoclonal antibody directed to a C-terminal epitope of collagen VII; d DAPI; **E** overlay of **A – D**. **(F–J)** skin thin section of a healthy 84 year old proband; immunofluorescent stains as in **A** through **D**; J overlay of **F – I**. **(K–O)** skin thin section of a 25 year old RDEB patient; ; immunofluorescent stains as in **A** through **D**, arrows point to the basal lamina where the collagen VII stain would be expected; O overlay of **K – N**. Please see text for an interpretation of this figure.

RDEB is caused by mutations in the *COL7A1* gene, which codes for the so-called anchoring fibrils that connect the basal lamina of the skin to the underlying dermis [6]. Therefore, to confirm the clinical diagnosis of RDEB in the studied patients, skin cryosections of the patients and healthy controls were analyzed for expression and correct localization of type VII collagen by immuno fluorescence microscopy (Fig.2). While we detected positive staining for type VII collagen directly below the epidermis in the skin of all healthy controls, type VII collagen staining was absent in all RDEB patients when the monoclonal antibody was used, confirming the diagnosis of severe RDEB [7].

However, we argued that the *COL7A1* mutations could all produce C-terminally truncated forms of the protein which would not be recognized by the mouse monoclonal antibody used which is directed to a C-terminal epitope. For this reason, we also incubated the patients' skin cryosections with a polyclonal rabbit antiserum, which could in principle also recognize more C-terminal epitopes. Indeed, this experiment showed faint collagen VII immunostaining (SI Fig. 1). The truncated protein, even if present in a small amount, could contribute to the pathological changes in this patient's skin.

### Genes coding for proteins of the cornified envelope

The genes discussed here were previously known as coding for proteins that are exclusively or pre-dominantly found in skin, are expressed in keratinocytes starting at various points during the life history of a keratinocyte and are finally cross-linked and found in cross-linked form in the cornified envelope (CE). Ultrastructurally, the CE is not dramatically changed, neither in skin of the elderly nor in RDEB skin (see Fig. 2). Still, the transcripts listed in this group show a rather dramatic change in abundance as determined by microarray analysis. In selected cases, transcript abundance data were confirmed by real time quantitative PCR methods as applied to the mRNA from young adult vs. elderly foreskin samples [8]. Where applicable, the quantitative PCR results were in line with the microarray data presented here (Table I). Protein visualization by immunohistochemistry on the respective thin sections was performed for loricrin, keratin 16, and collagen VII. In the cases investigated, it was found that the mRNA quantifications show the same trend as the protein data.

We are showing here that in whole genome microarray investigations of the skin samples from RDEB patients, we found significant increases and decreases of transcripts which are similar to the aging-specific ones. Table SI1 shows the complete list of genes in the overlap and lists the fold over- and under-expression values observed. The primary defect in the generalized severe RDEB skin is absence or extreme reduction of collagen type VII, which is normally located in the basement membrane zone, while the proteins of the CE are expressed at late stages of the keratinocyte maturation. First, we must therefore assume that the absence of collagen VII indirectly (perhaps through signaling mechanisms) leads to expression changes in these genes. Second, we observe that compensatory mechanisms probably act on skin of the elderly and likewise on RDEB skin, which can, for instance, compensate the loss of loricrin which we observed, by overexpression of other components of the CE. This is probably the reason why some of the major components of the CE are strongly down-regulated, while some others are up-regulated.

The components of the CE that were differentially regulated in the overlap list are described in detail now:

#### Loricrin

**Figure 3:**
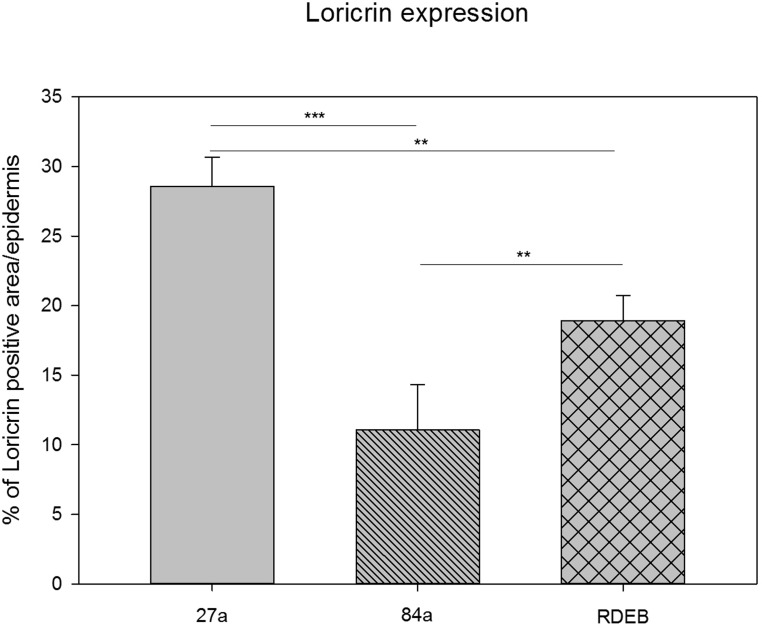
Quantification of loricrin immunoreactivity by using a particle size measurement tool revealed the highest loricrin expression in the epidermis of the middle aged proband (27a). A significantly reduced loricrin expression was detected in the aged proband (84a) as well as in the RDEB patient's epidermis.

This major component [9] of the CE, is cross-linked by transglutaminases. Its transcript is downregulated 21 fold in RDEB and six-fold in aging. Quantification of the immunofluorescence data for loricrin shows the same trend (Fig.2, Fig.3). The function and interaction of loricrin with other proteins of the CE is discussed in detail below.

#### **SPRR**s (small proline rich proteins)

There are 10 SPRR proteins encoded in the epidermal differentiation complex (EDC), three of them (SPRR1A/2D/2G) are substantially overexpressed in our overlap list (Table I). The SPRR proteins are small modules that share homology with loricrin (LOR). They are directly cross-linked with LOR by transglutaminases. The cross-linking reaction is the same as between LOR molecules, namely formation of a lysine to glutamine isopeptide bond, with loss of NH3. Little is known about functional diversification of the 10 variant forms of SPRRs, or about their tissue specificity. It is plausible that LOR and SPRR together are needed for the barrier function of the CE and that the bulk of the CE can be formed (although perhaps in an imperfect way) by overexpression of SPRR, thus leading to an ultrastructurally near normal CE which has, however, a defect in its barrier function.

#### **LCE1B** (late cornified envelope protein 1B)

The EDC contains a cluster of 18 *LCE* protein coding genes [10]. These genes are expressed in the latest stage of keratinocyte maturation. They are (like the *SPRR* genes) cross-linked into the CE and probably needed for the barrier function of the CE. One of those genes (*LCE1B*) is strongly overexpressed in both aging and RDEB. We propose that similar to the *SPRR* genes this is due to a hypothetical compensatory mechanism triggered by the reduction of loricrin expression observed here.

#### S100proteins

These are a large family of calcium binding and signal tranduction proteins with functions in many different human tissues. A subset of S100 proteins has also been found in skin and for some of these proteins including S100A7, their cross-linking into the CE [11–13] has been proven recently.

In the overlap list, no S100 proteins are down-regulated, but five proteins/genes are significantly up-regulated: S100A12, S100A7 (psoriasin), S100A7A, S100A8, S100A9 (Table I). These five proteins interact with RAGE (receptor for advanced glycation endproducts), an inflammation marker, and are all encoded in human chromosome 1q21 [14]. Interaction with RAGE occurs on the cell surface [15]. S100 proteins can be externalized by an alternative pathway different from the standard secretory pathway, but this question is not definitely clarified. Although RAGE expression or activity has been correlated with aging [16, 17], the gene (official gene name: *AGER*) is not in the overlap list. Furthermore, it is neither shown as differentially expressed in aging, nor in RDEB. The gene could be constitutively expressed in skin and the important regulation could be achieved by ligand binding. It is an important question whether the overexpressed RAGE-binding S100 proteins are functionally important in skin aging and RDEB through binding to RAGE or through other mechanisms. In the former case, what are the other components of the RAGE signaling pathway? In this context, p21RAS, MEK, and MAPkinases activating NF-kappaB were shown to interact with RAGE [18], however it is unknown whether they are activated in aging and RDEB.

Ca^++^ ions play a major role in keratinocyte maturation and in the formation of the CE [19] and one example is provided by the fact that Ca^++^ is an activating co-factor for the essential transglutaminases cross-linking CE proteins [9]. The five S100 proteins of skin which are overexpressed in aging and RDEB could play a role as Ca^++^ ligands in the pathogenic process. Ca^++^ concentrations in skin aging were determined by fluorescence microscopy in thin sections of foreskin and found to be substantially changed in aging [8, 20]. While in young adult skin, a calcium ion gradient was found with a peak in the stratum granulosum (where most of the cross-linking activity is believed to take place), in aged skin this gradient was shown to collapse. Until now it is unknown if intracellular calcium undergoes similar changes in RDEB.

The immunogenetics of asthma and eczema was studied and reviewed by Cookson [21]. The function of the S100 proteins of the skin [21] was found to be at least in part involved in the immune system of the skin. In particular, S100A2 is a chemotactic agent for eosinophils; S100A7 (psoriasin) is a chemotactic agent of CD4+ T cells and neutrophils with antibacterial activity. The S100A8 homodimer is a chemotactic agent for leukocytes, the S100A8-A9 heterodimer is a cytostatic inhibitor of macrophage activation and an inhibitor of Ig synthesis by B cells, and S100A12 is pro-inflammatory towards endothelial cells and is filaricidal and filaristatic.

Combining current literature on the S100 protein family members with the results shown in the overlap list, we can state that the up-regulation of the S100 proteins probably serves two purposes: First they are (in part) substrates for cross-linking and CE biosynthesis, and second they are signal transmitters via RAGE to induce and maintain the pro-inflammatory state of old skin and of RDEB skin.

### Genes coding for other proteins of human skin

Not surprisingly and considering the primary defect in RDEB patients in COL7A1, we also discovered transcriptional over- or under-expression of other skin-specific transcripts in the overlap list. They correspond to proteins found in all layers of the epidermis and in the basal lamina. As described below they represent minor variants of the respective protein families and in some cases have been associated with skin inflammation processes, which conforms to the clinical phenotype of the patients and to the aging theory known as „inflammaging“ [22, 23].

#### Integrin

One member of the integrin gene family is overexpressed three-fold in RDEB and four-fold in aging in human skin: integrin β6 (gene name *ITGB6*). All integrins act as heterodimers and constitute mechanical links functioning in signal transduction in both directions between epithelial cells and the basal lamina that surrounds every epithelial cell and other cells, for instance connective tissue cells [24]. The integrin heterodimers are therefore associated with hemi-desmosomes. In human skin, the most prominent integrin is α6β4. On the extracellular side it interacts with the very abundant laminin332, on the cytoplasmic side it interacts with talin, vinculin, and therefore indirectly with keratin intermediate filaments of epithelial cells. Hemidesmosomes are the anchoring points between the basal lamina and the epithelial cells (basal layer of keratinocytes).

In literature no prominent role is described for integrin β6 (*ITGB6*), located on chromosome 2q24 – q31, in healthy skin. Very few papers were published about *ITGB6* and skin [25–27], and its function in skin has not been fully elucidated yet. Nevertheless, the expression of *ITGB6* is induced by epithelial injury and participates in modulating epithelial inflammation [25]. Moreover, overexpression of human *ITGB6* in transgenic mice leads to chronic wounds and ulcers [26]. Furthermore, ITGB6 forms the αvβ6 heterodimer, which „is absent from healthy skin“[27] but induced in wound repair. This αvβ6 heterodimer activates TGF-β1, not cited in the overlap list.

Some forms of EB simplex are also caused by mutations in the *ITGB6* gene [7]. Interestingly, *ITGB6* is overexpressed in both differential gene lists (RDEB versus healthy and middle-aged versus aged skin), pointing to an important role of *ITGB6* overexpression in both of these situations. Consistently with this finding, as mentioned, *ITGB6* is induced by epithelial injury and plays a role in inflammatory processes which are important both in aging and RDEB.

#### Filaggrin

Filaggrin is a bundling protein for keratin intermediate filaments and therefore an important component of the architecture of healthy skin. Like other important proteins of the protective layers of the epidermis (like most prominently, loricrin), also filaggrin is strongly down-regulated on our overlap list. The two genes, FLG and FLG2, code for related but distinct isoforms of this structural component of the top layer of the epidermis. They are encoded in the „fused genes“ part of the epidermal differentiation locus on chromosome 1q21 – 22. The filaggrin locus on chromosome 1q21 – 22 is presently the most clearly recognizable genetic locus where mutations confer a predisposition for familial atopic dermatitis and asthma [28]. These mutations *per se* do not confer a severe loss of function of filaggrin [28] and in order to cause a full blown atopic disease presumably more mutations in unrelated genes (for instance genes of the immune system) as well as environmental factors are clearly needed. In contrast to this scenario, a severe loss of function of filaggrin leads to ichthyosis vulgaris, an autosomal semi-dominant inherited disease with high penetrance [29].

In order to fulfil its function in healthy skin, the high molecular weight profilaggrin has to be cleaved into protein monomers, which then associate with keratin intermediate filaments and with proteins of the cornified envelope [9, 29]. In this process, filaggrin is cross-linked into keratin bundles of the already enucleated corneocyte. Filaggrin also fulfils a second function in moisturizing the superficial layers of healthy skin, which is achieved by de-imination and breakdown to the constituent amino acids which accumulate to high millimolar concentration and are hygroscopic [9, 30]. Two filaggrin genes were downregulated in both, aged and RDEB patients:

##### FLG

This gene (synonym: *ATOD2*) encoding the major form of filaggrin in skin is 7-fold down-regulated in RDEB and 4-fold down-regulated in aging. In line with this, it is also strongly down-regulated in atopic dermatitis [31].

##### FLG2

This gene encoding an isoform of filaggrin is down-regulated 18-fold in RDEB and 5-fold in aging. *FLG2* function is distinct from *FLG* [32] in the process of cornification. It is a so-called S-100 fused type protein. Just like *FLG*, *FLG2* is markedly decreased at the protein level in atopic dermatitis, psoriasis, and more extremely in ichthyosis vulgaris, but increased in the hyperkeratoses.

This strong down-regulation of *FLG* and *FLG2* is certainly an important factor contributing to the pathogenesis of skin in aging and RDEB.

#### Laminin

Beside collagen IV, laminins are principal components of the basal lamina, a dense extracellular insulating structure which lies between the dermis and the epidermal cell layers. Laminins occur as heterotrimers and the genome contains genes coding for 5 α subunits, 3 β subunits and three γ subunits of laminin.

Linkage between the basal lamina and the basal keratinocyte layer is mediated by hemidesmosomes. The most important molecular link in this structure is between laminin 332 and the integrin heterodimer as has been described above (part on integrins). Obviously, balanced expression of the linker proteins is essential for the structural stability of the skin. As described above ITGB6 was found to be overexpressed in human skin aging and RDEB. In contrast to the overexpression of basal lamina associated proteins, we found that the laminin isoform LamB4 (***LAMB4*** coding for laminin subunit β4) is underexpressed two-fold in RDEB and 10-fold in aging skin.

The map location of ***LAMB4*** is 7q21.3 – 31.1, a candidate locus for dilated heart disease. Until now nothing is published about the specific function of this isoform in the skin. According to new data it is not a pseudogene as suggested previously [32]. As this isoform represents not an abundant component of the BL, the detected down-regulation in RDEB and aging is probably mechanistically unrelated to BL stability. Therefore, we can presently not suggest a direct functional link between LAMB4 underexpression and RDEB pathology.

#### Cystatin E/M *(CST6)*

The gene is down–regulated 2.5-fold in RDEB and 5.5-fold in aging. It is expressed in *stratum granulosum* in healthy skin and in *stratum granulosum* and *spinosum* in psoriasis. Cystatin E/M belongs to a superfamily of inhibitors of cysteine proteinases consisting of three subfamilies. Some proteins in the superfamily have lost the inhibitor function and fulfil other functions. *CST6* belongs to family II, which has 9 members, 7 of them on chromosome 20, and maps to chromosme 11q13 [33, 34]. It is expressed in differentiated epidermal keratinocytes and sweat glands [35] and is detected in tear fluid [36]. As Cystatin E/M targets proteases in skin [35, 37], its suggested role is in differentiation and cornification of keratinocytes in hair follicles and nails [38, 39].

The loss of this protein results in defects in the skin barrier function, as shown in the mouse [37] and in our patients, and in sweat glands and nails, as observed in RDEB and aging [40–42].

#### Keratins

We found that in the overlap list two of the keratins (cytokeratins) were strongly downregulated (*KRT2, KRT77*), while three were strongly up-regulated (*KRT16, KRT6A, KRT6B*). Considering the known evidence for the physiological function of these five genes in skin, we can show that these expression changes conform to the picture we have already gained. The epidermis in both pathological situations discussed here is thinner (smaller number of layers) and shows biochemical signs of chronic wounds and inflammation and activation of an immune response.

The human type I and type II keratin families and their clustered chromosomal locations on Chromosomes 17 and 12 were described by M.A. Rogers and colleagues [43, 44]. Intermediate filaments of the epidermal cells are built up by heterodimers of type I (acidic) and type II (basic) keratins and are differentially expressed in the different epidermal layers. During maturation of the keratinocytes the cells migrate into the superficial layers of the skin. In the special type of programmed cell death that occurs when the top layer of cornified cells is formed [45, 46], the CE is retained and is actually the main structure conferring the barrier function (partly mediated by lipids). However the keratin intermediate filaments are also retained and probably are responsible for the bulk of the structure, providing mechanical stability. The keratins making up this structure are cross-linked [45, 47] and are directly connected to desmosomes of keratinocytes.

The keratin isoforms KRT9, KRT10 (type I) and KRT1, KRT2 (type II) [9] are the major epithelial keratins, expressed in the granular and upper granular layer of the epidermis. These keratins are cross-linked to filaggrins and to themselves by transglutaminases [48, 49] and are responsible in part for the protection against water loss and for mechanical stability. Of the CE-associated keratins, only KRT2 is in the list of differentially regulated keratins in aging and RDEB. However, the strong overexpression of KRT16, which is not a major component in normal healthy skin, as well as the other gene expression defects described here (concerning, for instance, the CE components) may cause the imbalance in keratin expression described below.

KRT16 is shown to be strongly up-regulated in aged and RDEB skin (Table 1). It is a type I keratin encoded on human chromosome 17q12 – q21. This region contains K16 as well as K14 in several identical copies [50]. KRT16 is normally expressed in oesophagus, tongue and hair follicles and its expression has also been described in in epidermal keratinocytes [51]. KRT16 forms a heterodimer with K6, and both are strongly upregulated in the overlap list. Although KRT16 and K6A and K6B are defined as “epidermal” genes [52], their expression in epidermal keratinocytes is a marker of pathology. KRT16 is expressed in psoriatic skin and is an early marker of psoriasis. KRT16 and KRT6A mutations were described as causing pachyonychia (defect in hair and nails). KRT16 has a function in wound healing [53] and in regulation of the innate immune response in skin because it „regulates the innate immunity in response to epidermal barrier breach“[54]. Genetic control of KRT16 expression was studied by Chen [55]. Because KRT16 was found to be strongly overexpressed in RDEB and aging and was known to be a pathology marker, we decided to study it also at the protein level by immune fluorescence methods.

A possible hypothesis for the overexpression of the keratins K16, K6A, K6B in aged skin and RDEB skin, may be that they compensate for the extreme loss of loricrin.

***KRT6A***, a type II (basic or neutral) keratin, is up-regulated four-fold in RDEB and eight-fold in aging. It forms a heterodimer with K16 and was identified as the genetic locus of pachyonychia (see above). Six isoforms of *KRT6* were found at the human chromosome 12q12 – q13. The protein is mostly present in tongue, oesophagus, and oral mucosa and both in simple and stratified epithelial tissues.

The exceptional stability of the epidermal keratin architecture and the linkage of those keratins with desmoplakin of desmosomes is accomplished by keratins 1, 2, 5, and 6 [56]. Two of these four type II keratins were found to be strongly overexpressed in the present paper.

***KRT6B***, another basic or neutral type II keratin was found to be up-regulated 3.5-fold in RDEB and 6-fold in aging. Like *KRT6A*, it forms heterodimers with K16 and K17, is encoded on human chromosome 12q12 – 13, and is normally expressed in tongue, oesophagus, oral mucosa, hair follicles, and glandular epithelia.

***KRT2***, another basic or neutral type II keratin was found to be down-regulated six-fold in RDEB and 13fold in aging.

***KRT77***, another basic or neutral type II keratin, was found to be down-regulated 10-fold in RDEB and 25-fold in aging. Synonyms used in the literature are **K1b**, and *KRT1B*. This gene is expressed normally in eccrine sweat glands [57]. As mentioned above, a defect in sweat glands has been found in RDEB as well as in aging which confirms the present finding.

#### Collagens

All RDEB patients studied here carry a defect in the expression of *COL7A1* at the protein level, but overexpress the *COL7A1*-specific RNA about three-fold. The mutations in this group are loss-of-function recessive alleles. In our patients they are homozygous or compound heterozygous. We assume that the defect of collagen VII in some way triggers the extensive gene expression changes and phenotypes which we are observing here. It is therefore useful to summarize what is known about the function of *COL7A1* in human skin. The human genome carries 42 different independent loci coding for collagen alpha chains. These are very large genes with a high number of exons.

In the transcriptome overlap list (Table I) no collagen coding genes are down-regulated, but 4 different isoforms-coding genes are up-regulated (*COL13A1, COL1A1, COL4A2, COL6A3*). We will first give a short summary of *COL7A1* which is the mutated gene in the three RDEB patients and then in turn discuss the overexpressed collagen isoform encoding genes. A very useful overview of the human collagen protein family is given by Ricard-Blum [58].

##### COL7A1

This gene codes for the collagen type VII alpha chain. Synonyms found in the literature are *EBD1*, *EBR1*, and *EBDCT*. The collagen fibril is composed of three identical COL7A1 subunits (long chains), restricted to the basement membrane zone beneath stratified squamous epithelia. It forms the anchoring fibril between the basal lamina and the underlying dermal cells, probably via interaction with laminin 332 and type IV collagen [59] which in turn binds to dermal cell surface integrins [24]. It cooperates with the collagen IV network which is a major part of the basal lamina. This anchoring fibril is thought to be the major connection between the epidermis and the dermis and binds to fibronectin [60]. Note that fibronectin is not on the overlap list. Furthermore, RDEB patients present with a high incidence of cancers of the skin, typically squamous cell carcinomas. In line with this, *COL7A1* has been reported to possess also a signaling function in cancers of the skin [61].

##### COL13A1 [62]

A synonym found in the literature is *COLXIIIA1*. The gene is up-regulated about 2-fold in both aging and RDEB. It codes for a nonfibrillar collagen which is not secreted to the ECM, but localized as a trimer in the plasma membrane of connective tissue cells (fibroblasts) of nearly all connective tissues. The protein harbors a transmembrane domain. The gene contains eight exons and a complex splicing pattern results in a multitude of isoforms. However, no special function was so far discovered in the dermis.

##### COL1A1

A synonym found in the literature is OI4. The gene is nine-fold up-regulated in aging and four-fold in RDEB. It codes for the pro-α1 chain of the trimer formed by two α1 and one α2 chains. These proteins build up „collagen I“, which is the most abundant ECM protein in animals. It is abundant in bone, cornea, tendon and dermis. It is synthesised in dermal fibroblasts [63]. *COL1A1* mutations are found in osteogenesis imperfecta subtypes and in other inherited diseases. Overexpression of *COL1A1* leads to fibrotic diseases like the dermal fibrosis found in aging as well as in RDEB.

##### COL4A2

Synonyms found in the literature are *ICH* and *POREN2*. The gene codes for the α2 subunit of collagen IV and is overexpressed 5-fold in RDEB and three-fold in aging. The protein is part of the major protein network embedded in the basal lamina giving it strength interacting with integrin and the hemidesmo-somes and thereby supporting the strong interaction with the basal cells of the epidermis which is primarily through laminin of the basal lamina and integrin α4β6. It inhibits angiogenesis and tumor growth.

##### COL6A3

The gene codes for collagen VI, subunit α3 forming the „beaded filament collagen“ and is overexpressed three-fold in RDEB and two-fold in aging. The protein occurs in the ECM of nearly all connective tissues. Inherited diseases associated with mutations in *COL6A3* are Ullrich syndrome muscular dystrophy and Bethlem myopathy [64, 65].

### Genes involved in the immune response of human skin

This is the largest group of genes to be discussed here. Skin has an important immune function which works hand in hand with its function as a barrier against insults coming from outside, many of which are pathogenic microbes. It is known that this defense function is compromised in skin of the elderly but also in RDEB. We are showing here transcriptome data which support this notion and can also suggest a mechanism that results in a weakening of the defense function caused by structural changes due to gene expression changes of components of the epidermis (see above). Skin infections caused mainly by *Staphylococcus aureus* and *Pseudomonas aeruginosa* are frequent in RDEB [66]. Similarly, skin infections in the elderly are caused by *Streptococcus*, *Pseudomonas*, *Staphylococcus*, viruses (for instance Herpes) and fungi (for instance *Malassezia furfur*) [67]. Our data indicate that both the increased incidence of skin infections and the intrinsic pro-inflammatory state of skin in both aging and RDEB lead to the appearance of many genes on our overlap list which are related to the immune response. The intrinsic pro-inflammatory state may be present at the gene expression level even in the absence of strong signs of acute inflammation, which were absent in our aging skin samples. Concering the aging process, theoretical ideas have been put forward in the aging research community under the name of the „theory of inflammaging“ [22, 23], also in relation to skin aging [68]. The regulation of genes presented in the overlap list here strongly supports this theory.

Almost without exception, the genes codingα for components of the immune defense system which were found to be differentially expressed in both RDEB and aging, point to an increase in the activity of the immune response and the pro-inflammatory state. Genes which normally decrease the immune response through their activation, are found to be down-regulated, and genes which normally increase the immune response are found to be up-regulated.

### Overexpression of immune system genes

The first group of genes on the overlap list is induced by Interferon α (IFNα). These genes were found to be overexpressed in RDEB and aged patients, indicating an activation of an antiviral response [69].

#### 

##### IFI27

This gene is upregulated three-fold in RDEB and 10-fold in aging. It is normally induced by IFNα and known as a tumor marker [70], however, there is no published information on its function.

##### IFI44

This gene of unknown biochemical function is up-regulated 15-fold in RDEB and 6.6-fold in aging. It is induced by IFNα and after virus infection of cells and displays antiproliferative activity [71].

##### IFI44L

is induced three-fold in RDEB and four-fold in aging. It is a distinct isoform of IFI44, which has not been studied in detail so far.

##### IFIH1

This gene is induced two-fold in both RDEB and aging and is also known as MDA5 (melanoma differentiation associated gene 5). The gene is the locus for *Aicardi-Goutieres* syndrome. Gain of function mutations lead to up-regulation of type I interferon signaling. The protein is known to bind viral RNA and is induced after viral infection stimulating the autoimmunity response [72].

##### IFIT3

This gene of unknown function is induced three-fold in RDEB and two-fold in aging. It is induced by interferon alpha and contains tetratricopeptide repeats. IFIT3 induction is protective in virus infection of cells [69, 73, 74].

Another gene involved in antiviral response is also activated in the course of RDEB and aging and codes for the alpha subunit of IFNγ receptor (gene name *IFNGR1*).

##### IFNGR1

This gene is induced 4.5-fold in RDEB and 2-fold in aging. This receptor subunit is activated by virus infection and necessary for antiviral activity. Defects lead to severe immune deficiency concerning in particular immune activation of the T-cell response [75, 76].

#### Interleukins and interleukin receptors

##### IL13RA1

This gene coding for the alpha subunit of the receptor for IL 13 and IL4 is up-regulated 2.5-fold in RDEB and 2.7-fold in aging. It binds to the tyrosine kinase, TYK2, which is active in the JAK/STAT pathway. Activation of this gene in keratinocytes has been studied in asthma, psoriasis and atopic dermatitis. The protein is also expressed on mast cells [77]. These findings among others show similarities in gene expression between atopy, aging, and RDEB (see also the Conclusion part of this paper).

##### IL4R

This gene, encoding the alpha chain of the interleukin 4 receptor, is up-regulated 2.5-fold in RDEB and 3-fold in aging. Allelism studies show, that it is highly associated with asthma and atopy [78]. Ligand binding, expression, and activity of this receptor are important for atopy and asthma.

##### IL8

This gene is also known as *interleukin-8* or *CXCL8*. It is overexpressed 16-fold in RDEB and five-fold in aging. Expression of this gene is associated with cancer cell growth, and it is strongly pro-inflammatory [79]. This correlates well with the increased cancer incidence in RDEB and in aging [80–82].

Some members of the group of IL and IL-receptor genes are down-regulated but still conform well to the general picture of increased immune response in aging and RDEB (see below).

#### Defensins

##### DEFB124

This gene codes for defensin β124 and is up-regulated three-fold in RDEB and four-fold in aging. DEFB124 was found to be expressed in rat epididymis and testis, but until now no skin-specific expression has been described. DEFB124 belongs to the innate immune system and displays strong antibacterial action. Its up-regulation fits to the data presented here, highlighting a pro-inflammatory state of elevated antimicrobial activity in RDEB and aging [83, 84].

##### DEFB4

This gene codes for defensin β4 and is up-regulated 6.5-fold in RDEB and 39-fold in aging. *DEFB4* is a minor defensin in human skin, expressed only after infection or inflammatory reaction [85]. It is further expressed in cultured epithelial cells and *DEFB4* was shown to be regulated by NFκB [86].

#### Chemokines

The four overexpressed chemokines listed below were all described either for their role in cancer (leukemia), as leukocyte attractants to inflamed tissue or in early development. None of them is expressed appreciably in normal skin. Taken together, they supply further proof for the pro-inflammatory state of skin in RDEB and aging. Signaling through chemokines is complex and was reviewed recently [87].

##### CXCL12

This gene codes for the CXC chemokine ligand 12 and is also known as SDF-1 and PBSF [88]. It is overexpressed 2.6-fold in RDEB as well as in aging.

##### CXCL13

This gene codes for the CXC chemokine ligand 13 and is also known as *BCL, BCA, ANGIE, BCA-1, BLR1L, ANGIE2,* and *SCYB13*. It is overexpressed two-fold in RDEB and three-fold in aging. It is a B-lymphocyte chemoattractant, responsible for the homing of B lymphocytes to lymphoid follicles and a biomarker of inflammation. It is frequently expressed on tumors but has not been previously found in normal skin [87].

##### CCL2

This gene is located on human chromosome 17q, codes for the CC chemokine ligand 2 and is also known as HC11 and MCP-1. It is overexpressed 2-fold in RDEB and 3-fold in aging. The protein binds to chemokine receptors CCR2 and CCR4. It is secreted, processed and glycosylated, and up-regulated in psoriasis. It is a chemoattractant for monocytes and basophils that is active in skin [89, 90].

##### CCL5

This gene is located on human chromosome 17q in the same chemokine cluster as *CCL2*. It codes for the CC chemokine ligand 5 which is also known as RANTES and functions as chemoattractant for monocytes, eosinophils and Th cells. Multiple splicing variants exist which have not all been functionally characterized but do show anti-retroviral activity. Only a few papers deal with *CCL5* (RANTES) in skin, where expression is correlated with psoriasis and atopic dermatitis [91]. Overexpression of *CCL5* was not found in autoimmune EB [92] which is not surprising as in this form of EB no genetic defect is involved and the exact target of the autoimune antibodies is not known.

#### Complement

##### CFI

This gene, coding for complement factor I, is overexpressed three-fold in RDEB and four-fold in aging. It is also known as *FI, IF, KAF, AHUS3, ARMD13, C3BINA*, and *C3b-INA*. The gene product is a serine protease which is itself activated by proteolytic cleavage, disulfide formation (heterodimer) and glycosylation. It cleaves two downstream complement proteins: C4b and C3b, thereby inhibiting these complement components. The gene is essential for complement function and mutations lead to severe autosomal recessive immune deficiency. To summarize, the up-regulation of complement proteins in aging and RDEB is strongly indicated by the data presented here. In line with this, CFI was found to be over-expressed in SCC, a skin cancer frequently found in RDEB [93].

##### CD47

This gene is also known as *IAP, OA3*, and *MER6* and overexpressed about 2.5-fold in both RDEB and aging. The protein encoded by this gene is a cell surface membrane protein involved in calcium regulation after binding to the ECM, and is associated with integrin. CD47 displays a very broad tissue distribution, overexpression in aging [94] and interference with wound healing [95] has been reported thus conforming the picture of old and RDEB skin which we are presenting here.

#### Downregulation of immune system genes

A relatively small cohort of immune system genes is down-regulated in skin in aging and RDEB. Some of them are genuinely counteracting the up-regulation of an immune response which we generally observe, but some others are down-regulated thereby actually stimulating the immune response.

##### TNFRSF19

This gene codes for member 19 of the tumor necrosis factor receptor superfamily and is also known as *TAJ, TROY, TRADE*, and *TAJ*α. The protein is down-regulated 2.5-fold in RDEB and five-fold in aging. It is known to be overexpressed in embryonic development in skin and hair follicles [96] and can induce apoptosis by a non-classical mechanism. It is a biomarker for melanoma and cutaneous squamous cell carcinoma (SCC). Down-regulation of TNFRSF19 blunts the immune response.

#### Macrophage stimulating genes

##### MST1

This gene, also known as *HGFL*, codes for macrophage stimulating factor 1 and is down-regulated three-fold in RDEB and two-fold in aging. This serine/threonine specific protein kinase is a tumor suppressor which „promotes changes in the redox state by phosphorylation and inactivation of the peroxiredoxin-1 protein“ [97]. In other words, MST1 weakens the oxidative stress defense. Since the gene is down-regulated in RDEB and aging, the oxidative stress response is activated in these conditions. This is the only gene found so far in our overlap list (SI Table) that is functionally related to redox regulation. MST1 mediates apoptosis and interacts with GAPDH. It inhibits autophagy and shows sequence homology to *STE20*.

##### MSTP9

This gene is also known as *MST1L* and under 7 other synonyms. It is down-regulated 3.5-fold in RDEB and 3-fold in aging. The gene is generally considered to be a pseudogene located in the chromosome1 p36.2 „genomic graveyard“[98].

##### NFATC2

This gene is also known as *NFATP* and is down-regulated about 2-fold in both RDEB and aging. The protein encoded by NFATC2 is a nuclear factor of activated T-cells which is a calcineurin-dependent negative regulator of the immune response suppressing CD4^+^ T cells [99]. This means that the T-cell immune response is activated. It suppresses cell division via CDK4 [100, 101]. Interestingly, this is one example among others, where a member of a protein family which is normally not known as skin-specific still shows differential expression in RDEB and aging skin. Another member of the NFAT protein family, NFAT5, is indeed expressed in normal skin.

#### Interleukins (ILs) and interleukin receptors

Please note that other members of this group are up-regulated (see above).

##### IL1F7

This gene is located in the cytokine gene cluster on human chromosome 2 and was studied intensively resulting in 11 synonyms, one of which is IL37. It is down-regulated 3-fold in RDEB and 10-fold in aging. This interleukin is an inhibitory member of the IL family, binds to IL18 receptor β subunit and inhibits the immune response [102]. It suppresses pro-inflammatory cytokine expression and the innate immune response in psoriasis [103]. Expression of *IL1F7* is increased in atopic dermatitis [104]. It was described as a fundamental inhibitor of innate immunity [105]. In conclusion, the down-regulation of *IL1F7* is in accordance with the pro-inflammatory state and the increased immune response in old and RDEB skin.

##### IL1R2

This gene is located on human chromosome 2q12 and encodes for a soluble form of interleukin 1 receptor (a so-called decoy receptor) which is normally expressed in keratinocytes [106]. 7 synonyms exist in the literature, including IL1RB. The function of the protein is down-regulation of the immune response in skin by binding the ligand (IL-1) without creating signal transmission. It is down-regulated 3-fold in RDEB and 2-fold in aging. This result is in accord with a general pro-inflammatory and immune-stimulatory state in RDEB and aging.

##### IL22RA1

This gene is also known as CRF2-9 and is located in the cluster of interleukin receptors on on human chromosome 1p36. It is downregulated 2.4-fold both in RDEB and aging and encodes the interleukin 22 receptor subunit α1. The receptor for interleukin 22 acts together and shares subunit β with the IL10 receptor. The IL22 receptor is expressed on keratinocytes [107]. It is presently unknown how the down-regulation of *IL22RA1* fits into the general picture drawn for RDEB and aging.

### Ultrastructural investigation of the skin of young adults, elderly men, and RDEB patients

For each group of probands investigated here, immunofluorescence microscopy results are presented of one representative individual, which is similar in appearance to the other individuals. Thin sections of skin tissue were investigated by immunofluorescence for collagen VII (COL7), loricrin (LOR) and keratin XVI (KRT16). Keratin XVI was studied here because we saw the more than 10fold overexpression at the mRNA level in both aging and RDEB. Therefore, a mouse monoclonal antibody directed against collagen VII, a polyclonal rabbit antiserum directed against loricrin, a polyclonal goat antiserum directed against human keratin XVI, and 4′,6-diamidino-2-phenylindole (DAPI) for visualization of nuclear DNA were used. We are presenting the individual fluorescence images as well as the respective overlay of the four fluorescent stains.

Fig.2A - E: Foreskin thin section of a 27 year old healthy proband. Please note that the uppermost layer of the epidermis (*stratum corneum*) consists of a relatively small number (5–7) of layers of very flat cells that have already lost their nuclei through autophagy. This layer as well as cells of the underlying *stratum granulosum* (nuclei still present) show a strong immunoreactivity (IR) for loricrin. In addition, loricrin IR is weakly visible in all keratinocytes of the epidermis, where it is biosynthesized. Keratin XVI (KRT16) is nearly exclusively present in the keratinocytes of the *stratum spinosum*, which lie under the loricrin rich layers, and partially expressed in cells of the *stratum basale*. In healthy keratinocytes the expression of KRT16 is weak. Keratin XVI is described as a marker of inflammatory skin diseases, as mentioned above [54]. Collagen VII is synthesized by keratinocytes and fibroblasts in the skin and represents a major component of the anchoring fibrils, providing stability to the dermo-epidermal adhesion. Collagen VII IR is sharply localized to the dermo-epidermal adhesion site, beneath the *stratum basale* in the basal lamina. It is known from literature [58, 59] that it forms loops anchored in the lamina densa which provide mechanical strength by linking the basal lamina to the underlying dermis.

Fig. 2F - J: Foreskin thin section of an 84-year-old healthy proband. Here we discuss the obvious differences to Fig.2A with regard to morphology and immunofluorescence. The uppermost layer of keratinocytes (*stratum corneum*) consists of a somewhat smaller number of very flat cell layers, which in some cells seem to have retained the nucleus and are nearly completely devoid of loricrin. The remaining loricrin IR is detected mainly in the *stratum granulosum* but the total amount of loricrin seems to be lowered in aging (Fig.3), as stated previously [8]. Keratin XVI shows the highest expression in basal keratinocytes, directly neighbouring the basal lamina, while in Fig. 2A it seems to be more evenly distributed across the epidermal layers with the exception of the *stratum corneum*. It is difficult to judge the increase in protein level of keratin XVI in aging and RDEB from the immune fluorescence pictures alone. Previous work by our group showed that keratin XVI is overexpressed also at the protein level in aging skin using the western blot technique [108] and in EB simplex [109]. In the aged skin sample, collagen VII expression is not located strictly to the basal lamina, but is found more widely distributed in the extracellular space, extending more deeply into the dermis. One possible interpretation of this finding could be that it has to do with the presumed signaling function of collagen VII leading to the observed loss of loricrin and the structural changes in the upper layers.

Fig. 2K - O: Thin section of the skin of a 25-year-old RDEB patient. The striking similarity of this patient's skin with the aged skin shown in Fig. 2B lies mainly in the amount and distribution of loricrin. Loricrin is nearly absent from the *stratum corneum* and most of the remaining loricrin is located in the underlying *stratum granulosum*. Morphologically, at this level, the *stratum corneum* looks nearly normal with several cell layers of very flat cells without nuclei, however gene expression is different from wild type. This is also true for keratin XVI which is here clearly seen in keratinocytes in the *stratum basale*. Collagen VII is absent when tested with the monoclonal antibody used, which is a common trait for patients presenting with generalized severe RDEB [7] and is confirmed by our own clinical experience. As the monoclonal antibody is raised against an epitope at the C-terminus of type VII collagen, and the RDEB patients investigated here suffer from C-terminally truncated type VII collagen proteins, we additionally analyzed the expression of type VII collagen protein by immunofluorescence microscopy using a polyclonal serum. Application of the polyclonal serum (IgG fraction) visualized a faint type VII collagen labeling at the epidermal-dermal junction (SI Fig.1) that was not detected using the mouse monoclonal antibody. This means that C-terminally truncated collagen VII is still present in the RDEB patients, although at a low level. We speculate that the truncated protein could play an important pathophysiological role.

Fig. 3 shows a quantitative comparison of the loricrin immunofluorescence visible in Fig. 2A, B, and C. The decrease in loricrin in aging as well as in RDEB is highly significant and is even stronger in aging than in RDEB (One-way ANOVA, Bonferroni t-test). Comparing the percentage of positive loricrin particle size per epidermis, the loricrin expression in the RDEB patient (18.93%, SD: 1.8) is reduced compared to the 27-year-old healthy donor (28.54%, SD: 2.12), showing a mean difference of 9.61%. Compared to the 84-year-old healthy donor (11.07%, SD: 3.25), the mean difference of loricrin expression to that in RDEB is 7.86% and therefore smaller than in comparison to the young healthy donor. These data indicate similarities of the loricrin expression also at the protein level between aged and RDEB skin.

## CONCLUSION

In the present paper we have described and compared transcriptomic differences between young adult skin, skin of the elderly, and skin of severe RDEB patients and have also correlated the gene expression data with ultrastructural investigations of skin, emphasizing three antigens, collagen VII, loricrin and keratin XVI. These data complement each other and point to a previously unknown strong similarity between aged skin and RDEB-affected skin.

The mutant type VII collagen protein in each of the three patients investigated is a shortened form caused by chain termination. This protein appears to be absent when a monoclonal antibody directed to the C-terminus of the protein is used for detection, but is faintly visible when a polyclonal serum is used. The transcript corresponding to the protein is increased about three-fold in the patients. We speculate that these *COL7A1* RNAs or the shortened proteins could be causally involved in the disturbance of skin–specific gene expression which we observe.

In addition, the gene expression data presented here also showed an unexpected but strong similarity with the expression data known in psoriasis, asthma and atopy. Examples for genes where this correlation is striking are *IL13RA1* and *IL4R*, which are both overexpressed, and the subset of S100 proteins which we found to be overexpressed in RDEB and aging. In a very general way, we conclude that the pro-inflammatory state which we observed in RDEB and aging, is also similar to that in atopic diseases like asthma and atopic dermatitis.

Three important functional groups of genes were studied, which code i) for components of the CE, ii) for other parts of human skin, and iii) for the innate and adaptive immune system that is active in the skin. Genes in these groups show large changes in expression and can thus explain some of the clinical phenotypes.

Besides these three large groups of genes, we may mention other groups of genes which were not discussed in detail, for instance the proteases and protease inhibitors which are functional in skin biology and are found on the overlap list of 261 genes (see the Supplementary Table). In addition, we mention also genes coding for ion channels, for metabolic enzymes, and finally genes that are not yet functionally annotated but may be important for understanding the pathomechanisms discussed here.

One of the gene groups which is conspicuously absent from the overlap list is the group of genes of the skin redox biology and oxidative stress defense. This is surprising because a large number of genes in the human genome (probably several hundred) deal with defense against oxidative stress and it has been described that both the intrinsic skin aging process and the blistering diseases of skin involve an increase in oxidative stress and oxidative damage to skin [110–113]. We should stress here again that the skin samples used for the present work were not severely exposed to extrinsic aging factors like the ultraviolet part of sunlight. For extrinsic aging, oxidative stress is even more important than for the intrinsic aging process [110, 114]. It should be mentioned here that in the larger list (unpublished) of transcripts differentially expressed in aged skin from which the overlap list was derived, the functional groups of oxidative stress and mitochondria do occur. Among the 1440 transcripts in this list, for instance, transcripts corresponding to the quantitatively most important proteins of oxidative stress defense are found. One obvious (but unproven) tentative explanation for the absence of the same genes from the overlap list (SI Table I) could be that the response to oxidative stress in RDEB skin may be primarily regulated at the post-transcriptional level and is therefore not seen in the transcriptome changes discussed here.

Another surprisingly absent (with very few exceptions) group of genes concerns the genes of lipid metabolism. The barrier and defense function of the CE strongly depends on structural lipids, including the complex lipid, ceramide, and long chain hydroxylated fatty acids like ω-hydroxy ceramide [9]. Again, this aspect of the barrier function of skin may be controlled mainly at the post-transcriptional stage. Alternatively, it could be that the lipid components and their biosynthetic enzymes are not appreciably altered at the transcriptome level in skin aging and in RDEB. These questions remain open to future research. What we have shown here is a striking similarity in clinical phenotype and genome-wide transcriptome data between skin aging on the one hand and dystrophic epidermolysis bullosa on the other hand. At the gene expression level this similarity is most clearly seen in genes coding for structural and regulatory components of the skin, but also in genes coding for the immune response in a broad sense. A suprisingly large part of the skin components found to be differentially expressed are located in the cornified envelope.

Smoller et al. [82] discussed that alterations within the dermis might be responsible for an “activated immunophenotype” state of the epidermis in conditions that predispose to squamous cell carcinomas, including actinic keratoses, defects in wound healing, and RDEB, characterized by expression of involucrin, filaggrin, and other proteins. This hypothesis is supported by Ng et al. [81], who found that in RDEB skin, fibroblasts form an environment that is permissive for the development of aggressive squamous cell carcinomas in RDEB skin by enhancing both proliferation and invasiveness of neighbouring keratinocytes. Re-expression of the *COL7A1* gene in RDEB fibroblasts, which is defective in RDEB, reduces this phenotype. Therefore, the main drivers of SCC development in RDEB skin are probably not the pre-cancerous keratinocytes themselves, but the fibroblasts residing within the connective tissue supporting them [81]. A similar mechanism might also take place in aging skin, which also presents with an increased risk for SCC [80]. Therefore, the similarity in gene expression that we observed in RDEB-affected and aging skin might partly result from changes that lead to an “activated immunophenotype” in skin in both conditions.

The similarities between RDEB-affected and aged skin also implicate that the study of rare skin diseases may also yield novel insight into the mechanisms underlying common skin diseases and skin aging. Furthermore, it suggests that therapeutic strategies aimed at common diseases and conditions (such as skin aging) might also be used to alleviate the symptoms of rare diseases (such as RDEB) and vice versa.

## SUPPLEMENTARY INFORMATION, FIGURE



## Abbreviations

RDEB: recessive dystrophic epidermolysis bullosa
EB: epidermolysis bullosa
CE: cornified envelope
PBS: phosphate buffered saline
TBS: TRIS buffered saline
PFA: *para*-formaldehyde
RT: room temperature
BSA: bovine serum albumin
RGB: + SPRR: small proline rich proteins
EDC: epidermal differentiation complex
LOR: loricrin
LCE: late cornified envelope
LCE1B: late cornified envelope protein 1B
RAGE: receptor for advanced glycation end products
ITGEB6: integrin beta 6
TGF-beta1: transforming growth factor beta 1
FLG: filaggrin
FLG2: filaggrin family member 2
LAMB4: laminin subunit beta 4
BL: basal lamina
CST6: cystatin E/M
KRT16: keratin 16
KRT6A: keratin 6A
KRT6B: keratin 6B
KRT2: keratin 2
KRT77: keratin77
IFNalpha: interferon alpha
IFNGR1: alpha subunit of interferon gamma receptor
IFI: group name of genes which are induced by interferon alpha
MDA5: melanoma differentiation associated gene 5
IL13RA1: alpha subunit of the receptor for interleukin 13
IL4R: alpha chain of the interleukin 4 receptor
IL8: interleukin 8
DEFB124: defensin beta 124
DEFB4: defensin beta 4
CXCL12: CXC chemokine ligand 12
CXCL13: CXC chemokine ligand 13
CCL2: CC chemokine ligand 2
CCL5: CC chemokine ligand 5
CFI: complement factor I
SCC: squamous cell carcinoma
CD47: cluster determinant 47 (a cell surface protein of the immune system)
TNFRSF19: member 19 of the tumor necrosis factor receptor superfamily
MST1: macrophage stimulating factor 1
MSTP9: nuclear factor of activated T cells C2 IL1F7: interleukin 1 F7 (inhibitory member of the interleukin family)
IL1R2: interleukin 1 receptor 2 (a soluble decoy receptor)
IL22RA1: interleukin 22 receptor A1
IR: immunoreactivity
