# Supplementary material for: Transcriptome and ultrastructural changes in dystrophic Epidermolysis bullosa resemble skin aging
Source: Aging (Albany NY). 2015 Jun 14;7(6):389–411. doi: 10.18632/aging.100755 (PMC4505166; doi:10.18632/aging.100755)
Supplement: Supplementary file 1 [file aging-07-0389-s001.pdf]

SUPPLEMENTARY INFORMATION

Please browse the full text version of this manuscript to see the Supplementary Table.

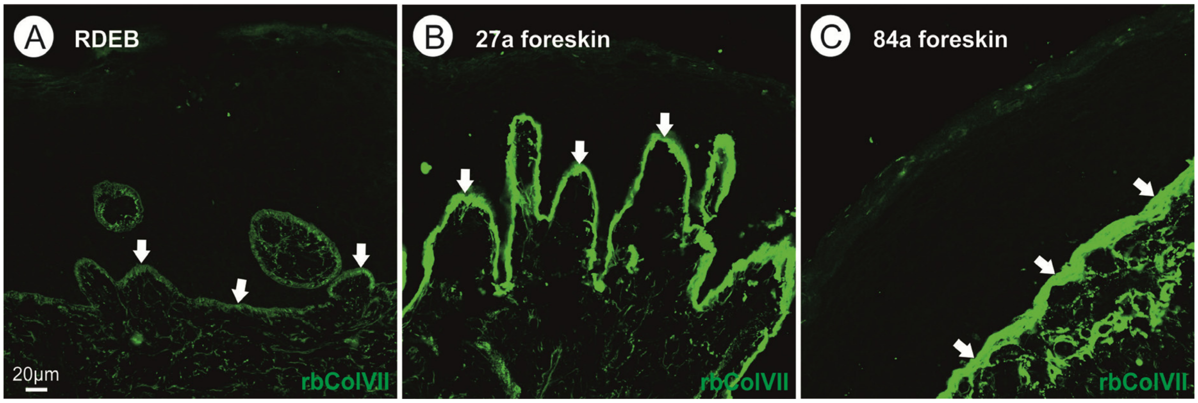

Supplementary Figure 1. (**A**) Detection of type VII collagen protein with the rabbit polyclonal anti type VII collagen serum visualized a faint type VII collagen labeling at the epidermal-dermal junction (arrows) in RDEB patients compared to a strong immunoreactivity in (**B**) the middle aged and (**C**) aged skin tissue. Please compare this result with Fig. 2M and see discussion in text.
